# Monitoring health inequality in Indonesia

**DOI:** 10.1080/16549716.2018.1475041

**Published:** 2018-11-26

**Authors:** Ahmad Reza Hosseinpoor, Devaki Nambiar, Anne Schlotheuber

**Affiliations:** Department of Information, Evidence and Research, World Health Organization, Geneva, Switzerland; George Institute for Global Health, Delhi, India

**Sekali merengkuh dayung, dua tiga pulau terlampaui**.

***With one stroke of the paddle, two– three islands have passed.***

This is a common saying in Indonesia, suggesting that in one action, multiple goals may be achieved. It is perhaps a fitting metaphor for those who see the sweeping breadth and scope of the Sustainable Development Goals (SDGs) not as a challenge, but as an opportunity. Our experience shows that the SDGs are indivisible, intersectional and synergistic. For instance, the health goal SDG 3 puts at its heart the notion of universal health coverage, which is premised on the idea of ensuring equity. This is enabled by SDG Target 17.18 which calls for data disaggregation as a means of both monitoring and tackling inequality. Further, SDG 10 on reducing inequalities is also served by the purposes of improved data systems, and efforts to achieve universal health coverage.

One such stroke of the paddle in Indonesia, as it were, began in 2016, resulting in the country’s first comprehensive assessment of health inequality, published in December 2017 [1]. Over 50 indicators across 11 health topics were disaggregated by dimensions of inequality, such as household economic status, education level, place of residence, age or sex. Apart from this process of collaboration, capacity-building, analysis and advocacy at the national level, those involved with the process were also committed to dissemination in the academic community, manifest as nine distinct contributions in this special issue on health inequality monitoring in Indonesia.

Health inequality monitoring – in Indonesia or in any other setting (at global, regional, national or subnational level) – adds an equity dimension to standard health monitoring. This is done by ensuring the availability and use of disaggregated data on population subgroups of interest. The process of health inequality monitoring is a continuous cycle comprising five steps: the selection of health indicators and dimensions of inequality; the sourcing of this data; its analysis using both disaggregated data estimates and summary measures of inequality; reporting of results; and the implementation of policy and/or programme changes in light of findings.

The first paper in this special issue highlights the 20-month capacity-building process of health inequality monitoring in Indonesia, which led to the development of myriad products including a national state of health inequality report and several publications [2]. It outlines the capacities required for health inequality monitoring and then details the content and duration of the process in Indonesia, describing stakeholders (including the lead agency and contributors), outcomes, successes, challenges and lessons learned. It also takes a view on the adaptation and implementation of analogous processes of health inequality monitoring in other countries.

A critical initial step towards creating a system for health inequality monitoring, which includes collecting, analysing and reporting health inequality data, is mapping available data sources. A short communication outlines the process by which data source mapping was undertaken in Indonesia, presenting in detail the template used for this purpose, as well as various data sources from Indonesia identified through its use (e.g. the Census, the Basic Health Research Survey, and various topic- and disease-specific surveys, as well as the sample registration system and health facility data) [3]. Availability of disaggregated data on particular health topics was mapped across 16 of these sources in order to determine which source would be best suited for use in the creation of Indonesia’s national state of health inequality report.

Another essential ingredient for health inequality monitoring, one that allows customisation and visualisation of inequality analyses, is HEAT Plus. HEAT Plus is a software application developed by the World Health Organization (WHO) to facilitate analysis and reporting of health inequality data [4]. HEAT Plus is the upload database edition of the Health Equity Assessment Toolkit [5]; it allows users to upload data on any indicator disaggregated by any dimension of inequality following a simple template. In Indonesia, HEAT Plus was used for the analysis of inequality across 11 health topics, some of which are included in this special issue.

One of the papers employing HEAT Plus examines data from the National Socioeconomic Survey on access to improved drinking water and sanitation, disaggregated by district within Indonesia’s provinces using both absolute and relative inequality measures (i.e. mean difference from mean and weighted index of disparity) [6]. Large variation in inequality was observed within provinces with the same numbers of districts. The province of Papua, for instance, stood out in that it had among the lowest levels of coverage in the country (only 28% of the population reporting access to improved sanitation), as well as the highest levels of inequality (a value of 92.3% for its weighted index of disparity as compared to 3.1% in DKI Jakarta), clearly indicating domains and locations of priority.

A unique contribution to this special issue is one describing analysis of subnational regional inequality in the form of Indonesia’s Public Health Development Index (PHDI), a composite of 30 indicators across several health topics with 7 sub-indices (for reproductive and maternal health, newborn and child health, infectious diseases, non-communicable diseases, environmental health, health risk behaviours and health service provision) [7]. This index was developed for use in priority-setting, planning and resource allocation across districts in the country. For this special issue, both average levels of PHDI and within-province inequalities were reported. Provinces in western Indonesia tended to report higher overall PHDI scores than those in the east. Cases were also identified where within-province inequality in PHDI and its sub-indices were higher in provinces with the same average scores, especially in the case of the environmental health index, again suggesting domains of priority for addressing inequality.

In the course of using HEAT Plus, there was discussion about Indonesia’s high rates of adolescent tobacco use, relative to other countries. This topic was delved into more deeply using logistic regression methods on data from the 2013 Basic Health Research Survey [8]. It was found that the odds of current smoking were higher among males and older adolescents, and among those in the poorest quintile. Prevalence also tended to be significantly higher in the western provinces as compared to those in the east, controlling for individual socio-economic and demographic characteristics. The article discusses how prevalence may be shaped by historical factors and the broader policy context related to tobacco, pointing towards areas for programme and policy action.

Moving from analysis to action, a methods article in this special issue lays out the process to increase the equity-orientation of health programme and policy workflows in Indonesia [9]. The WHO’s Innov8 approach comprised eight steps that sought to understand the design and programme theory of these plans, develop consensus around who is being left out by these plans and why, and develop a redesign proposal that foregrounds intersectoral action and social participation, while also ensuring monitoring and evaluation. The first review of the Maternal Health Action plan led to the generation of various recommendations. To bring focus, a training session for district health authorities was developed in order to inform their annual planning cycles in 10 provinces. Concurrent demand for capacity to analyse, report and visualise data on inequalities was met with the introduction of HEAT Plus for health inequality monitoring, described at length in this special issue [2].

Many of the processes followed, as well as challenges and opportunities observed in Indonesia, are applicable to other countries and settings, even as they may be at different stages of development in terms of their national health information systems. The debate article on health inequality monitoring highlights common challenges across countries in the domain of data collection, analysis, reporting and effective communication, as well as the development of strategies to address inequalities identified [10]. It also describes shared opportunities, including broad-ranging global initiatives that aim to standardise data collection, its analysis and/or also to improve the quality and level of customisation of communication, as well as guidance on how equity-oriented policymaking can occur. The WHO in particular has developed tools and resources to support these elements of health inequality monitoring [4,11–15].

The second debate article showcases WHO’s broad strategy for Gender, Equity and Human Rights to which health inequality monitoring is a major contributor [16]. It presents definitions of key concepts and principles in this domain – equity, gender and the right to health. It also briefly describes other components of WHO’s work, including: barrier analysis in Nigeria using qualitative approaches; human rights monitoring in coordination with the United Nations High Commissioner for Human Rights; guidance provided to Member States through WHO country offices under the National Health Policies, Strategies and Plans (NHPSPs), citing the case of Mongolia in particular; and programme reviews using the Innov8 approach, with application to Nepal’s Adolescent Health strategy. The broader goal for WHO has been and will continue to be to ensure country adaptation, moving away from single-method, siloed and exclusively technocratic approaches that may have been relied upon in the past.

Findings across papers converged, perhaps indicative of the demographic and epidemiologic transition underway within Indonesia, requiring prioritisation of provinces and districts for particular health issues and dimensions of inequality. The province of Papua, for instance, appears to have the greatest within-province inequalities in terms of sanitation as well as the PHDI, while adolescent smoking is not a priority area in this province. Prevalence rates of smoking were significantly higher in western provinces, and among poorer adolescent males. Thus, in making the effort to understand health inequalities in Indonesia, the country has used one paddle to pass many islands. The process brought certain issues to the fore (like adolescent smoking), showcased unique analytics, and highlighted indicators related to health as well as its determinants (like water and sanitation). More broadly, the process of capacity-building for health inequality monitoring in Indonesia documented here is a rough template for adaptation and customisation to improve the use and equity-orientation of other national health information systems. This can be facilitated by a growing armamentarium of tools the WHO has developed to support health inequality monitoring at the global level and in countries. There are clear synergies with policymaking and lessons for other members states wishing to make great strides – or in this case strokes! – on the path to leaving no one behind.
